# Oxidative Stress and Redox Modulation Potential in Type 1 Diabetes

**DOI:** 10.1155/2011/593863

**Published:** 2011-05-18

**Authors:** Meghan M. Delmastro, Jon D. Piganelli

**Affiliations:** ^1^Diabetes Institute, Division of Immunogenetics, Department of Pediatrics, Children's Hospital of Pittsburgh, University of Pittsburgh School of Medicine, Pittsburgh, PA 15224, USA; ^2^Department of Immunology, University of Pittsburgh School of Medicine, Pittsburgh, PA 15224, USA; ^3^Rangos Research Center, Department of Pediatrics, Children's Hospital of Pittsburgh of UPMC, 4401 Penn Avenue, Pittsburgh, PA 15224, USA

## Abstract

Redox reactions are imperative to preserving cellular metabolism yet must be strictly regulated. Imbalances between reactive oxygen species (ROS) and antioxidants can initiate oxidative stress, which without proper resolve, can manifest into disease. In type 1 diabetes (T1D), T-cell-mediated autoimmune destruction of pancreatic **β**-cells is secondary to the primary invasion of macrophages and dendritic cells (DCs) into the islets. Macrophages/DCs, however, are activated by intercellular ROS from resident pancreatic phagocytes and intracellular ROS formed after receptor-ligand interactions via redox-dependent transcription factors such as NF-**κ**B. Activated macrophages/DCs ferry **β**-cell antigens specifically to pancreatic lymph nodes, where they trigger reactive T cells through synapse formation and secretion of proinflammatory cytokines and more ROS. ROS generation, therefore, is pivotal in formulating both innate and adaptive immune responses accountable for islet cell autoimmunity. The importance of ROS/oxidative stress as well as potential for redox modulation in the context of T1D will be discussed.

## 1. Introduction

Oxidation-reduction or redox reactions are pivotal to maintaining life through respiration, metabolism, and energy supply. Mitochondria, which are known to be the powerhouses of the cell, possess the ability to utilize nutrients to generate energy (redox potential) via the electron transport chain, which donates electrons to oxygen to yield ATP and H_2_O [1, 2]. Consequently, oxygen free radicals, known as superoxide (O_2_^−^), are nonenzymatically leaked from the mitochondria and react with other molecules to create reactive oxygen species (ROS) such as hydrogen peroxide (H_2_O_2_), peroxynitrite (ONOO^−^), and hydroxyl radical (OH^−^), all of which can alter DNA, proteins, carbohydrates, and nucleic acids [3–5] and may eventually lead to irreversible damage. The inability of a cell's antioxidant defenses to overcome oxidative injury and accretion of severe ROS-mediated damage over time will eventually lead to cell death [5–7]. In order to maintain a reduced environment, several cellular antioxidant defenses are in place, including glutathione, glutathione peroxidase, catalase, and three different superoxide dismutase (SOD) enzymes: SOD1, 2, and 3, located in different subcellular and extracellular locations. A basal level of “accidental” superoxide is accumulated in healthy individuals [1, 8], which has been widely hypothesized to be responsible for aging and the associated pathologies [9–11]. However, oxidative stress occurs from an imbalance between ROS and antioxidant actions. During chronic oxidative stress caused by environmental factors (i.e., UV light, ionizing radiation, toxic substances), infections, or lack of dietary antioxidants, an inequity of cellular reducing equivalents capable of detoxifying the increased burden of ROS has marked effects on normal cellular processes. However, in times of oxidative stress, normal cellular respiration is also still functioning, resulting in dysregulated mitochondrial free radical production and disparity between ROS generation and antioxidant defenses [6, 12]. The combination of stress-induced and conventional mitochondrial dysfunction can manifest into disease states, including cancer [13–15], rheumatoid arthritis [16, 17], neurological disorders [18–21], pulmonary diseases [22], and type 1 diabetes [23–26]. 

## 2. Redox and Inflammation

What once was thought to be solely derived from the mitochondria, reactive oxygen species have now been shown to be produced by an important family of primarily immune system-associated enzymes [27–29]. The NADPH oxidase (NOX) family of enzymes is designed to combine NADPH and oxygen to actively generate superoxide. Activated phagocytes, such as macrophages, monocytes, and dendritic cells (DCs), as well as neutrophils, form ROS within the phagosomal membrane for efficient killing of a wide array of invading pathogens [30]. The protection afforded by the phagocytes is crucial, but not without side effects. Production of highly permeable reactive oxygen species (i.e., H_2_O_2_) causes leakage of these molecules from phagocytes and therefore, unwanted effects on bystander cells [31, 32]. In an environment high in oxidative stress, these bystander reactions drive increased activation of the immune system, cell damage, and progression to disease. For example, NOX-derived ROS have been shown to stimulate mitogenic signaling and proliferation [33, 34], which can have potential deleterious consequences on the promotion of tumorigenesis [35, 36] and in the context of autoimmunity, can lead to T cell expansion [37]. Additionally, H_2_O_2_ can augment monocyte chemokine receptor surface expression critical for migration to sites of infection and inducing inflammation [38] as well as can promote VEGF signaling to trigger angiogenesis, with implications in cancer and tumor progression [39]. Furthermore, ROS generated from both mitochondria and NADPH oxidase complexes can also act intra-cellularly as well as inter-cellularly as signal transduction molecules. Hydrogen peroxide has been suggested to inactivate protein phosphatases [40], as well as to activate protein tyrosine kinases [41, 42] and metalloproteases through the oxidation of critical cysteine residues [43, 44]. Phosphatases such as SHP-1 serve to decrease inflammation by inhibiting tyrosine kinase activity, yet this type of regulation is lost upon cysteine oxidation [45–48]. Similarly, latent metalloproteases require oxidation for activation and, in the presence of hypochlorous acid (HOCL) and H_2_O_2_, secretion of chemotactic mediators (L-selectin and proinflammatory TNF*α*) is highly increased [49], thus enhancing inflammation. In addition, H_2_O_2_  has been demonstrated to freely cross the plasma membrane and activate NF-*κ*B, a redox-dependent transcription factor [50, 51]. NF-*κ*B plays a major role in immunity by promoting proinflammatory cytokine production, cell proliferation, and inflammation. In general, receptor-ligand interactions are known to generate ROS [52, 53]. In the immune system specifically, LPS interaction with Toll-like receptor 4 (TLR4) has been shown to facilitate the binding of TLR4 to NADPH oxidase 4 (Nox4) and subsequently release ROS [54], resulting in the activation of NF-*κ*B and generation of proinflammatory cytokines IL-1*β* and TNF*α* [53]. In a highly oxidized environment, the binding of pathogens to innate cell receptors can lead to hyperresponsiveness [55], suggesting inflammation is secondary to oxidative stress [25, 56]. Not only are phagocytic cells critical for early pathogen recognition through receptor-ligand interaction, they are also necessary for activation of the adaptive immune response. Following antigen recognition by phagocytic antigen-presenting cells (APC), an adaptive immune response is acquired in secondary lymphoid organs through synapse formation of APCs with lymphocytes, as well as from critical innate-derived ROS and third signal proinflammatory cytokines (TNF*α*, IL-1*β*) enhancing T-cell activation, proliferation, and effector function [37, 57]. Within this interaction, the H_2_O_2_ made by the phagocytes is able to traverse the synapse and act upon the T cells, at concentrations ranging from 10–100 *μ*M [58, 59], resulting in a feed-forward mechanism stimulating T-cell-specific NF-*κ*B activity and subsequent proinflammatory cytokine production. Similar effects of ROS are also seen on B cells [60]. Moreover, antigen stimulation of the TCR also drives endogenous production of H_2_O_2_  through the T cell's own NOX enzyme [28, 61]. Intracellular H_2_O_2_ can then signal and lead to T-cell proliferation, apoptosis [61, 62], and in conjunction with proinflammatory cytokines, promote T-cell effector function [37, 52, 63]. Therefore, in the presence of oxidative stress, an inability to balance the oxidation with antioxidant enzymes can drive chronic inflammation from both the innate and adaptive arms of the immune response [64], manifesting into many clinically relevant diseases, particularly type 1 diabetes. 

## 3. Oxidative Stress and Type 1 Diabetes

Type 1 diabetes or insulin-dependent diabetes mellitus (T1D) is an autoimmune disorder involving immune-mediated recognition of islet *β*-cells by autoreactive T cells, which leads to the liberation of ROS and proinflammatory cytokines, resulting in the destruction of pancreatic *β*-cells in the islets of Langerhans and loss of insulin secretion. Patients with T1D must constantly prevent hyperglycemia by administering exogenous insulin or in the situation of severe hyperglycemic unawareness, by undergoing islet transplantation. Despite a multitude of efforts in trying to specify the exact etiology, the cause of T1D is still under debate. The combinatorial effects of genetic susceptibility, environmental factors, and dietary deficiencies are known to contribute to disease origin; however, the impact of oxidative stress in a genetically susceptible individual is of particular interest. Oxidative stress, as stated above, occurs when the generation of ROS overcomes the scavenging abilities of antioxidants. Such instances may be mediated by genetic lack of antioxidant enzymes as well as environmental triggers like viral infections. Overall, oxidative stress has been linked to *β*-cell cytotoxicity [65–67] and has been suggested to play a role in T1D pathology [68–71]. Several studies show that the total serum antioxidant status, as measured by urate, Vitamin C, and total plasma antioxidant levels, of prediabetic and T1D patients is lower in comparison to age-matched controls [72, 73], which inevitably leads to greater oxidative modification of proteins and lipids [74]. Other literature illustrates a connection between viruses, ROS production, and type 1 diabetes onset. Gamble et al. demonstrated a positive correlation between type 1 diabetes onset and Coxsackie B4 virus infection through antibody titer measurements [75, 76]. Furthermore, such infections have been shown to cause indirect [77] and direct *β*-cell damage [78] and to stimulate the *β*-cells into secreting inflammatory mediators themselves [79]. ROS are made following viral infection from activated phagocytes [80, 81], as mentioned previously, and work to not only cause cellular injury but also can activate inflammatory, redox-dependent transcription factors, such as NF-*κ*B, perpetuating inflammation. Viral-mediated ROS production or a reduction in antioxidants can have severe consequences as *β*-cells are more prone to oxidative damage than most other tissues. The *β*-cell mitochondria have exceptionally low levels of glutathione peroxidase, superoxide dismutase, and catalase activity [24, 82–84]. Because of this low antioxidant defense, *β*-cells can be clearly disrupted by oxidative stress and, in genetically predisposed individuals, results in easy targets for a subsequent cytokine-mediated autoimmune attack.

Mitochondrial and NOX-derived ROS both have implications in *β*-cell destruction and T1D. Increased glucose causes rapid induction of the tricarboxylic acid (TCA) cycle within the *β*-cell mitochondria, which can lead to augmented ROS production [85]. The superoxide leaked from mitochondria can then form H_2_O_2_ and work to uncouple glucose metabolism from insulin secretion [86]. Ultimately, high levels of mitochondrial ROS can cause *β*-cell death [87, 88]. Intriguingly, models of T1D induce disease by generating toxic amounts of ROS within the islets (i.e., streptozotocin and alloxan) [89]. Alloxan is easily taken up by *β*-cells [90], where it is reduced into dialuric acid and subsequently reoxidized to establish a redox cycle [91]. ROS generated by alloxan treatment have been shown to promote islet *β*-cell DNA fragmentation, culminating in cell death [92]. In contrast, an alloxan-resistant strain of mice, the ALR mouse, shows increased ROS dissipation and resistance to islet destruction [23, 93, 94], further implicating the importance of oxidative stress and T1D. Streptozotocin (STZ), on the other hand, causes *β*-cell DNA alkylation and eventually drains the cellular NAD^+^ and ATP source in an effort to repair the DNA [95]. Xanthine oxidase is then able to utilize dephosphorylated ATP as a substrate for superoxide production [96]. Additionally, STZ metabolism increases the levels of islet cell nitric oxide (NO) [97], which together with superoxide can generate peroxynitrite (ONOO^−^). Detection of peroxynitrite in prediabetic nonobese diabetic mouse (NOD) islets suggests importance of this ROS in *β*-cell death [71]. Similarly, NOX enzymes have been detected within the pancreatic *β*-cells [98, 99]. Hyperglycemia can increase the assembly of NOX enzymes through its p47phox subunit, and therefore, enhance superoxide generation [100] and facilitate *β*-cell death. 

## 4. Immunology of T1D

Autoimmune diabetes onset is preceded by infiltration of immune cells into the pancreatic islets. Ultimately, a breach in tolerance to self-antigens allows for autoreactive T cells to become activated and attack the *β*-cells, resulting in the loss of insulin secretion. However, innate immune cells, such as macrophages and DCs, are of the first cells to enter the islets during insulitis [101, 102]. Although resident macrophages are present in the pancreas at all times, acquisition of antigen is required for macrophage activation and the production of cytokines. As described above, genetic and environmental factors can lead to cell destruction, releasing *β*-cell-specific antigens as well as ROS [103]. Macrophages will phagocytose dying *β*-cells and present antigen in the context of their MHC molecules. In humans, specific HLA molecules HLA-DR3 and DR4 are correlated with a susceptibility to T1D [103, 104]. Moreover, the ROS created by the initial insult to the islets are able to stimulate the activation of redox-dependent NF-*κ*B and other transcription factors within the macrophages [105]. Activated macrophages secrete a mixture of proinflammatory cytokines such as TNF*α*, IL-6, IL-1*β*, and ROS, which can start to damage the pancreatic *β*-cells [106–108]. IL-1*β* can cause extensive cytolysis in *β*-cells [109] through the upregulation of iNOS and subsequent generation of nitric oxide (NO) [110, 111], whereas TNF*α* enhances IL-1*β*-mediated islet destruction and helps activate APCs and T cells [112–114], but does not cause direct *β*-cell apoptosis *in vivo* [114]. 

**Figure 1:**
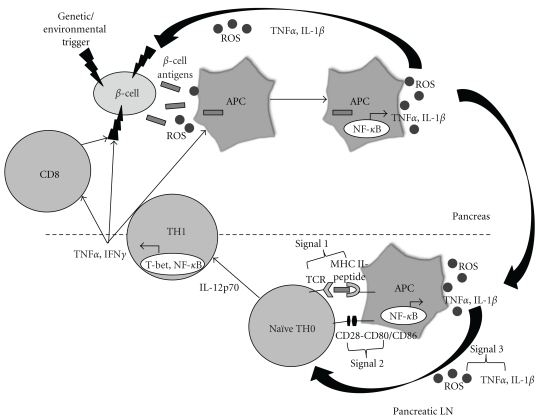
*Role of redox in the immunopathology of type 1 diabetes*. An initial genetic or environmental insult to the beta cell triggers the release of beta cell antigens as well as the production of ROS. Beta cell antigens are phagocytosed, and ROS are able to stimulate redox-dependent transcription factors such as NF-*κ*B, which leads to APC activation and cytokine secretion. ROS and proinflammatory cytokines secreted by APCs act as the third signal within the T-cell-APC immunological synapse, which occurs in the pancreatic lymph node. ROS play a critical role in the progression of naïve TH0 cells to cytokine-secreting TH1 cells. Release of IFN*γ* by TH1 cells then works directly on the beta cells as well as activates more APCs and CD8 cells, all of which can impart deleterious effects on the islets.

ROS and cytokines released by APCs not only promote *β*-cell damage, but also help to generate an adaptive immune response, which in T1D, is the crucial step in autoimmune destruction. It is well established that chronic elicitation of antigens to innate immune cells in a highly oxidized environment will lead to MHC-peptide presentation, perpetuating an adaptive immune response [115, 116] (Figure 1).

In the context of continuous *β*-cell ablation, macrophages can phagocytose dying cells and migrate to the pancreatic lymph node where they interact with naïve T-cells. It is this aforementioned synapse that enables T-cell proliferation and effector function to occur. In the presence of all three necessary signals: (1) MHC-peptide, (2) costimulation, and (3) soluble third signal, in this case consisting of ROS, IL-1*β*, and TNF*α*, T cells become activated via NFAT and NF-*κ*B [117–121]. Furthermore, IL-12 released from macrophages can differentiate CD4^+^ T cells into the TH1 lineage via signaling through STAT4 [122–125]. CD4^+^ TH1 cells then home to the site of antigen production, the *β*-cells, and call in other T cells and more APCs through the secretion of IFN*γ*. IFN*γ* has some indirect effects on *β*-cells, including potentiating the maturation of pancreatic APCs, which can then elicit an even greater T-cell response [126]. Additionally, neutralization of IFN*γ* in NOD mice has been shown to reduce both diabetes and insulitis [127], whereas IFN*γ*R-deficient NOD mice demonstrate delayed insulitis, but do not develop T1D [128]. Proinflammatory cytokines TNF*α*, IL-1*β*, and IFN*γ* all play a role in *β*-cell death primarily through activation of redox-regulated transcription factors NF-*κ*B and STAT1 [129–131]. Combinations of TNF*α* with IFN*γ* or IL-1*β* are necessary for primary murine *β*-cell death [132], and TNF*α*/IFN*γ* act synergistically to activate the stress-activated proapoptotic JNK/SAPK pathway, which promotes *β*-cell apoptosis via p53 and intracellular ROS [133]. The activation of NF-*κ*B can also increase iNOS and Fas expression, potential inducers of cell death, while downregulating the antiapoptotic Bcl-2 gene [134]. Apoptosis of *β*-cells is also mediated partially by T-cell expression of Fas ligand, TNF*α*, and perforin/granzyme [114, 134]. Specifically, CD4^+^ T cells are thought to be sufficient for T1D onset [135, 136], whereas CD8^+^ T cells seem to play a lesser role in the final stage of autoimmune destruction [137]. It is known, however, that synergy between both CD4 and CD8 T cells results in absolute transfer of diabetes in rodent models [138, 139]. Although specific to the model of autoimmune diabetes, TNF*α* secretion from CD4^+^ T cells can activate TNFR1 on *β*-cells and cause apoptosis [140], while CD8^+^ T cells can kill NOD *β*-cells by a Fas-dependent mechanism [141] or by perforin release [142]. Ultimately, T-cell exacerbation of *β*-cell death comes from endogenous generation of ROS and cytokines following APC activation [143] that can perpetuate islet destruction through a feed forward mechanism. Overall, ROS are crucial in not only activating the initial infiltrating macrophages and DCs [144] via the common denominator NF-*κ*B, but also for subsequently driving an adaptive TH1 immune response that is necessary for total ablation of *β*-cells and progression to T1D [134, 136]. Therefore, therapies would be most beneficial if there was not only protection of the *β*-cells from ROS, but also inhibition of the ROS-mediated autoimmune attack, possibly by preventing NF-*κ*B activation, ensuing inflammation, and the initiation of the adaptive immune response. 

## 5. Controlling Redox in T1D

Glutathione peroxidase (GPX), superoxide dismutase (SOD), and catalase are categorized as the most crucial antioxidant enzymes; however, islets inherently contain only a fraction of the enzymatic activities in comparison to liver, which possesses the highest abundance [145]. Because of the low antioxidant defenses present in pancreatic islets, therapeutic strategies to enhance antioxidants and reducing capabilities are of utmost importance. Studies utilizing overexpression of GPX1, SOD1 (Cu/Zn SOD), SOD2 (MnSOD), or SOD mimetic administration in insulinoma cell lines such as NIT-1 and INS-1 afforded protection from ROS and reactive nitrogen species (RNS) *in vitro* [25, 146, 147]. Usage of SOD mimetics in other inflammatory models has also demonstrated diminutions in proinflammatory cytokines [148, 149]. Furthermore, stable transfection of insulin-producing RINm5F cells with GPX, catalase, and Cu/Zn SOD resulted in defenses against cytokine toxicity imparted by the combination of IL-1*β*, TNF*α*, and IFN*γ* [150]. Antioxidant overexpression has been linked to not only protection against ROS and cytokines, but also to enhanced cell proliferation and decreased death. PDX1, a transcription factor necessary for *β*-cell differentiation, survival, and insulin synthesis [151], is also very responsive to ROS [152], where high oxidation causes a cytoplasmic relocation of PDX1 out of the nucleus, increased degradation of the protein, and subsequent dysfunction of *β*-cells [153, 154]. By alleviating ROS within the islets, PDX1 protein has exhibited stability and enhanced function in type 2 diabetes models [155], which can also have implications in T1D for stabilizing *β*-cell function and survival. Other experiments utilize transgene or adenoviral technology to overexpress antioxidant genes within the *β*-cells to specifically show islet-mediated versus autoimmune protection from T1D. These studies have elicited conflicting results. For example, overexpression of metallothionein and catalase in *β*-cells was unable to delay or inhibit spontaneous diabetes onset within NOD mice and promoted reduced activation of the PDX1 survival pathway [156]. Metallothionein proteins are intracellular, cysteine-rich molecules with high redox potential [157]. Similarly, transgenic expression of extracellular SOD in *β*-cells does not confer any difference in T1D incidence in comparison to control NOD mice [158]. These results suggest that basal levels of ROS production are necessary for *β*-cell function, possibly by triggering appropriate insulin signaling and regulating cell survival [159]. In contrast, overexpression of thioredoxin, a redox-regulated protein which helps repair ROS-damaged proteins and DNA, constitutes protection of *β*-cells from autoimmune and STZ-induced diabetes [160]. *β*-cell-specific transgenic expression of catalase and metallothionein is also able to shield isolated islets from hydrogen peroxide and reduce the effects of STZ treatment [161–163]. Transgenic expression of heme oxygenase-1, which has crucial cytoprotective functions against oxidative stress and inflammation, can improve insulitis and spontaneous diabetes in NOD mice [164], and alloxan-induced diabetes is also reduced following overexpression of Cu/Zn SOD in *β*-cells [165]. Moreso, precedence for the importance of enhancing islet-associated antioxidant levels has been demonstrated at the genetic level, in which mice resistant to alloxan treatment (ALR mice) exhibit protection from diabetes [94, 166]. This finding particularly helps further justify the need for therapeutic discovery and necessary experiments to determine druggable targets based upon modulation of antioxidant function. 

Systemic administration of antioxidants, in comparison to overexpression studies, shows more consistency in ameliorating T1D. Administration of 16 mg/kg/day of a potent antioxidant to young NOD mice resulted in a reduction of diabetes incidence from 89% in controls to 44% in the treated animals [167]. Furthermore, after a multiple low dose administration of STZ, addition of zinc sulphate to the drinking water of animals was able to increase metallothionein levels, inhibiting the onset of T1D [168], whereas intraperitoneal injections of butylated hydroxyanisole (BHA) antioxidant were able to attenuate the production of proinflammatory cytokines by islets and macrophages, thereby lowering insulitis and hyperglycemia [169]. Such uniformity in these results versus the transgenic expression of multiple antioxidants, as discussed above, may relate to the ability of systemic therapies to not only protect the *β*-cells but to also inhibit immune system activation and inflammation. Adenoviral delivery of systemic heme oxygenase to NOD mice decreased insulitis and T1D incidence; however, this alleviation was associated with a decrease in mature DCs and TH1 effector function [170]. Additionally, ALR mice resistant to alloxan-induced diabetes contain specific genetic modifications conferring systemic elevation of antioxidants, resulting in neutrophils with reduced superoxide bursts [171]. In an *in vitro* system using the antioxidant probucol, which can delay alloxan-induced [172] and spontaneous diabetes in rats [173], macrophages exhibit decreased H_2_O_2_ production, thus maintaining islet viability [174]. Further reports on the effects of systemic antioxidants on innate immunity include studies from our lab utilizing metalloporphyrin-based catalytic antioxidants (CA) with bone marrow-derived macrophages. The CA houses a metal center that catalyzes superoxide dismutation, mimicking SOD activity [175, 176], and is able to scavenge a broad range of ROS including O_2_^−^, H_2_O_2_, ONOO^−^, and lipid peroxyl radicals [53, 177, 178]. Following treatment with CA and LPS stimulation of macrophages, the production of nitrite (NO_2_^−^), O_2_^−^ TNF*α*, and IL-1*β* was significantly reduced in comparison to control [25, 53]. This effect was mediated by the ability of CA to oxidize the p50 subunit of NF-*κ*B within the nucleus, inhibiting its binding to DNA and subsequent transcription of proinflammatory cytokines [53]. Redox modulation of transcription factor DNA binding has previously been demonstrated for NF-*κ*B as well as other eukaryotic molecules [179, 180]. Inhibition of NF-*κ*B has been well established as an effective method of thwarting the immune response and resolving inflammation to maintain *β*-cell integrity [181, 182]; however, we are the first to illustrate a link between metalloporphyrin catalytic antioxidants, blockade of NF-*κ*B activation, and delayed autoimmune diabetes, as described below.

**Figure 2:**
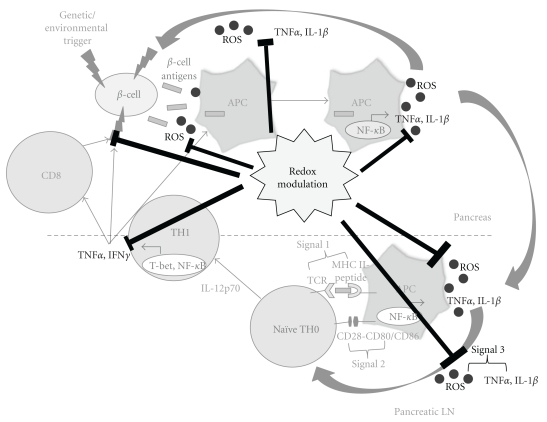
*Role of redox modulation in controlling ROS-mediated beta cell destruction*. Redox modulation has shown promise in blocking the production of ROS and its ability to activate APCs, resulting in diminished TH1 cell activation and effector function, which ultimately may help regulate beta-cell destruction.

 The activation of macrophages and T cells relies on oxidative stress, which ultimately leads to the progression of T1D. Based upon this fact, CA was also investigated in the context of CD4 and CD8 T cells. The BDC-2.5 TCR-Tg TH1 cell clone, which has recently been described as specific for the protein ChgA, a member of the granin family of neuroendocrine secretory proteins [183], causes rapid transfer of diabetes into NOD.*scid* recipients [184]. By utilizing this method, pretreatment of NOD.*scid* mice with CA prior to adoptive transfer of the BDC-2.5 clone inhibits the infiltration of T cells into the pancreas, significantly delaying T1D onset. Moreover, APC-dependent BDC-2.5 T cell proliferation and IFN*γ* production are also reduced after *in vitro* CA treatment [25]. To further delineate the mechanism of diminished T-cell effector function, *in vivo* treatment of NOD and BDC-2.5 TCR-Tg mice with CA was able to decrease innate-derived third signal synthesis, primarily consisting of TNF*α*, resulting in antigen-specific T cell hyporesponsiveness [37]. Similar results were found upon CA treatment in the context of CD8 T cells, reducing proliferation, cytokine production, and cytolytic effector molecules of CTLs [185]. Interestingly, by inhibiting NADPH oxidase in NOD animals (NOD.*Ncf1^m1J^*) in an effort to genetically mimic systemic CA administration, not only is NOX-derived superoxide production eliminated, but T cells show reduced TH1 responses, granting protection from T1D onset [120]. Earlier studies by Chaudhri et al. supported our experimentation by demonstrating attenuation of T-cell proliferation and IL-2R expression following antioxidant treatment [186, 187]. Such findings point to the possibility and importance of redox modulation in not only regulating the innate immune cells, but also impacting the T cells which formulate an adaptive immune response crucial for the autoimmune attack in T1D (Figure 2).

In addition to decreasing oxidative stress imposed on the islets, which can directly damage *β*-cells or indirectly stimulate the autoreactive immune response to become activated, redox modulation may also be useful for decreasing the unyielding ER stress within the *β*-cells. Because the *β*-cells are a constant source of insulin and insulin must be folded properly for secretion, the importance of balancing a high protein-folding load with survival of the cells increases substantially in comparison to other nonsecretory cells [188]. An overload of misfolded proteins may eventually result in cell death, if not properly resolved. An early study by Lo et al. highlighted the susceptibility of *β*-cells to ER stress by overexpressing MHC class II proteins in islets, essentially overwhelming the protein folding machinery and leading to apoptosis [189]. Other more recent studies show biochemical connections between ER stress-induced apoptosis and *β*-cell death, through both calcium-dependent and independent molecules [190–192]. To reconcile protein misfolding within the ER, the unfolded protein response, or UPR, is consequently triggered [193, 194]. The UPR acts as a backup mechanism to protect cells from accumulating unfolded proteins and to restore the balance between the protein folding machinery and the secretory pathway [195]. However, an accumulation of unfolded proteins during severe ER stress is sometimes unable to be resolved by the UPR, as characterized in the Akita mouse which contains a mutation in the proinsulin 2 gene that disrupts insulin folding, retains it within the ER, activates UPR, yet still eventually leads to *β*-cell death [196, 197]. Moreover, ROS have been suggested in supporting the UPR towards a more proapoptotic than proadaptive level [198], further illustrating the importance of regulating oxidative stress to maintain *β*-cell survival. Although the UPR paradoxically utilizes an oxidative environment within the ER to correctly fold proteins (i.e., disulfide bond formation), sustained oxidative stress can perpetuate the UPR to a level that promotes apoptosis [198, 199]. Additionally, the abundance of ROS present during continued unadapted ER stress can trigger apoptosis in neighboring cells as well. This is especially critical in islet *β*-cells, where their ability to handle oxidative stress is already reduced because of low levels of antioxidants [24, 82–84]. More pertinent is when unresolved ER stress leads to dying *β*-cells containing the misfolded proteins. These cells can be taken up by resident pancreatic APCs and presented to autoreactive T cells within the pancreatic lymph nodes. This type of event may stimulate the reactivity of T cells to formerly tolerated “neo-autoantigens,” which can ultimately promote more *β*-cell destruction and eventual development of autoimmune diabetes [200, 201]. A study conducted by Malhotra, et al. shows that antioxidant treatment of CHO cells results in not only decreased oxidative stress, but also decreased misfolded proteins, reduced activation of the UPR, and enhanced secretion of proteins [188]. Thus, it appears that a temporal or redox balance is essential for optimal *β*-cell function. In situations where the *β*-cell may experience environmental stressors that lead to disruption of the ER-machinery, the results may set in motion both ER-stress-induced UPR and the expression of misfolded proteins in an oxidative environment, further providing an optimal milieu for driving autoreactive T cells to become activated. Therefore, redox modulation may serve yet another purpose: to help reduce ER stress and subsequently maintain *β*-cell viability. 

Although the ability to predict susceptibility to type 1 diabetes is becoming increasingly accurate [202], and therefore, prophylactic treatment of patients with antioxidant therapeutics is not out of the realm of possibilities, currently a more feasible option for individuals with chronic hyperglycemia is to undergo islet transplantation. Islets, like any other transplantable organ, are in short supply; however, maintaining function and viability of transplanted islets is the major drawback of the procedure [161]. Not only are islets susceptible to immune rejection, but hypoxia during isolation and transplantation is the primary cause of *β*-cell death [203]. Because of their low resistance to ROS [24, 82–84], *β*-cells are especially vulnerable to oxidative damage and ischemia-reperfusion injury [204, 205]. In order to combat this weakness, the application of antioxidants seems a suitable alternative, as they have shown promise in liver and kidney transplantations [206, 207]. Longer allograft survival times have been demonstrated with mouse islets soaked with hydroxyl-radical inhibitors prior to transplantation [208] and with multiple *in vivo* administrations of SOD and catalase prior to and after islet transplantation [209]. Likewise, transduction of islets with heme oxygenase-1 or SOD2 genes was able to improve viability and insulin secretion *in vitro* [210] and elicit greater functionality upon transplantation in comparison to controls [211], respectively. Furthermore, we have also demonstrated benefit using the catalytic antioxidant approach, whereby adding CA during and after human islet isolation enhanced cell survival and function, allowing for normalization of STZ-induced diabetic NOD.*scid* mice [212]. Additionally, CA is not only able to protect human islets from STZ cell damage, but can also protect murine islets from both antigen-independent innate-mediated inflammation and antigen-dependent T-cell-mediated allograft rejection [204]. Overall, unlike common antirejection drugs, which are outstanding at protecting against the adaptive immune response but fail to shield islets from ROS/inflammation [213, 214], our CA treatment is nontoxic to islets and can alleviate both the alloimmune [204] and autoimmune responses [25, 37, 53, 185]. 

## 6. Conclusion

Although redox has been extensively studied in the context of both T1D and type 2 diabetes [85, 215], the plethora of literature discussed above shows the implications of ROS in all stages of autoimmune T1D, including the primary “trigger”, the initiation of insulitis by the innate immune system, and the acquisition of T-cell-mediated autoreactivity. These studies open the door to novel ideas of redox modulation, such as targeting ROS-dependent immunological metalloproteases [43, 44, 49] or disrupting the autoreactive T-cell pool, as described [37, 120, 185]. Moreover, a study evaluating self-antigen-primed T cells demonstrates how NO is able to reduce FOXP3 expression and subsequently decrease Tregs in autoimmune disorders [216], illustrating how intricate and vast the role of redox is in the immune response and where future studies may focus. In addition to effects on the target organ(s) and the immune system, autoimmunity also gives rise to systemic problems, and in the context of diabetes, ROS have been characterized as crucial elements promoting hyperglycemia-induced diabetic complications, especially those involving the vasculature [6, 217]. One important study conducted by Ling et al. provided evidence of oxidative stress-mediated vascular complications in prediabetic NOD mice [218], which exemplifies the importance of ROS in not only exacerbation of disease, but also on initiation of T1D and nonhyperglycemic associated pathologies. Furthermore, utilizing antioxidants, such as Vitamin E, cannot only assuage vascular activation [219], but can also grant protection from the loss of secondary target organ function, such as the kidneys [220]. Therefore, oxidative stress affects every aspect of T1D and the benefit of redox modulation may be more important than once thought. Optimal treatments may have to incorporate antioxidants with anti-inflammatory agents, such as inhibitors of NF-*κ*B activation, and must also take into consideration the limitations associated with utilization of intact enzyme/protein therapies, including bioavailability, immunogenicity-limited cellular accessibility, and cost of production. However, with the advent of newer nonpeptidyl small compounds, alleviating oxidative stress through antioxidant therapy appears to be a plausible druggable target. This therapy should restore balance between oxidation and reduction, leading to resolution of inflammation, thus reducing the autoimmune destruction of the islet *β*-cells.
